# Rosuvastatin Attenuates Vascular Dysfunction Induced by High-Fructose Diets and Allergic Asthma in Rats

**DOI:** 10.3390/nu16234104

**Published:** 2024-11-28

**Authors:** Elena-Larisa Zimbru, Răzvan-Ionuț Zimbru, Valentin-Laurențiu Ordodi, Florina-Maria Bojin, Daniela Crîsnic, Minodora Andor, Silvia-Nicoleta Mirica, Ioan Huțu, Gabriela Tănasie, Laura Haidar, Daciana Nistor, Luminița Velcean, Virgil Păunescu, Carmen Panaitescu

**Affiliations:** 1 Center of Immuno-Physiology and Biotechnologies, Department of Functional Sciences, “Victor Babes” University of Medicine and Pharmacy, 300041 Timisoara, Romania; elena.zimbru@umft.ro (E.-L.Z.); razvan.zimbru@umft.ro (R.-I.Z.); valentin.ordodi@upt.ro (V.-L.O.); florinabojin@umft.ro (F.-M.B.); crisnic.daniela@umft.ro (D.C.); gtanasie@umft.ro (G.T.); daciana_nistor@umft.ro (D.N.); vpaunescu@umft.ro (V.P.); cbunu@umft.ro (C.P.); 2 Research Center for Gene and Cellular Therapies in the Treatment of Cancer—OncoGen, Timis County Emergency Clinical Hospital “Pius Brinzeu”, No. 156 Liviu Rebreanu, 300723 Timisoara, Romania; 3 Chemistry and Engineering of Organic and Natural Compounds Department, University Politehnica Timisoara, 300006 Timisoara, Romania; 4 Discipline of Medical Semiotics II, Department V—Internal Medicine—1, “Victor Babes” University of Medicine and Pharmacy, 300041 Timisoara, Romania; andor.minodora@umft.ro; 5 Multidisciplinary Heart Research Center, “Victor Babes” University of Medicine and Pharmacy, 300041 Timisoara, Romania; 6 Faculty of Sport and Physical Education, West University of Timisoara, 4 Vasile Parvan Bd., 300223 Timisoara, Romania; nicoleta.mirica@e-uvt.ro; 7 Horia Cernescu Research Unit, Faculty of Veterinary Medicine, University of Life Sciences “King Michael I of Romania”, 300645 Timisoara, Romania; ioan.hutu@fmvt.ro; 8 Cardiology Clinic of the Timisoara Municipal Clinical Emergency Hospital, 12 Revolution of 1989 Bd., 300040 Timisoara, Romania; lumyciunguvelcean@gmail.com

**Keywords:** inflammation, allergic asthma, vascular dysfunction, cardiovascular disease, rosuvastatin, organ bath, vascular reactivity

## Abstract

Background: A growing body of evidence links a high-fructose diet (HFrD) to metabolic disturbances, including inflammation, dyslipidemia, insulin resistance and also endothelial dysfunction, yet its role in allergic asthma remains underexplored. Considering that obesity and hypercholesterolemia exacerbate asthma by promoting systemic inflammation, investigating interventions with dual metabolic and anti-inflammatory effects is essential. This study aimed to evaluate the potential modulatory effects of rosuvastatin in ameliorating the effects of HFrD-induced metabolic and vascular dysfunction in the context of allergic asthma. Methods: Forty-eight Sprague-Dawley rats were assigned to eight groups, receiving either a standard or HFrD for 12 weeks. Allergic asthma was induced using an ovalbumin sensitization and challenge protocol, while controls were administered saline. Selected groups were treated with rosuvastatin throughout the entire duration of the experiment. Body weight, abdominal circumference and serum biomarkers were assessed at baseline, 6 and 12 weeks. Endothelial function was assessed by evaluating vascular reactivity in an isolated organ bath. Additionally, histopathological analyses of aortic and pulmonary tissues were conducted to investigate inflammatory responses and morphological changes. Results: Rats on HFrDs exhibited significant increases in body weight, abdominal circumference, lipid profiles and blood glucose, which were further aggravated by allergic asthma. Rosuvastatin treatment notably reduced lipid levels, C-reactive protein and immunoglobulin E, while also enhancing vascular reactivity and attenuating aortic and bronchial wall thickening. Conclusions: Our findings suggest that rosuvastatin may serve as an effective therapeutic agent for addressing vascular and inflammatory complications associated with a high fructose intake and allergic asthma.

## 1. Introduction

The burden of cardiometabolic syndrome associated with dietary patterns is substantial in both developed and developing nations, primarily emerging from a sedentary lifestyle and an imbalance between caloric intake and expenditure [1,2]. Despite a worldwide decrease in fat consumption in the past decades, the intake of added sugars has significantly increased [1]. While fructose was firstly deemed as an ideal substitute for glucose in the diet of diabetic patients due to its non-stimulatory effect on insulin secretion, studies have associated high fructose consumption with insulin resistance, dyslipidemia, endothelial dysfunction, obesity and inflammation [3,4]. The significant rise in the prevalence of diabetes, obesity and coronary heart disease in the last decades also correlates with the exponential increase in fructose consumption [5,6].

Allergic asthma ranks among the most prevalent chronic inflammatory conditions, with both obesity and elevated serum cholesterol levels identified as contributing risk factors for its development [7,8,9,10,11]. There have been reported ultrastructural changes in mitochondria in the lungs of mice fed with a high-fructose diet (HFrD), even without allergen exposure [12]. The high fructose/glucose ratio in sweetened beverages, fructose malabsorption and the formation of asthma-triggering immunogens in the gastrointestinal tract lumen have also been linked to asthma [13,14,15].

Individuals with allergic asthma displayed greater carotid intima–media thickness and elevated levels of inflammatory biomarkers compared to those without asthma [16,17,18,19].

Given that both hyperlipidemia and asthma involve inflammatory processes, there is a possibility of pathophysiological interactions between these two conditions [16,20,21,22,23]. However, further exploration is needed to understand the inflammatory connection between allergic asthma and atherosclerosis.

The composite lipid parameters, like atherogenic index (AI), atherogenic index of plasma (AIP), lipoprotein combination index (LCI), Castelli risk index I (CRI I), Castelli risk index II (CRI II) are currently used to assess cardiovascular risk more effectively than traditional single lipid measurements. They combine multiple lipid components to provide a more comprehensive evaluation of an individual’s lipid profile and associated risk of cardiovascular disease [24,25,26,27].

Inhibitors of 3-hydroxy-3-methyl-glutaryl (HMG)-CoA reductase were discovered in the early 1970s, and now, half a century later, the anti-inflammatory and lipid-lowering attributes of statins have gained worldwide recognition in the prevention of cardiovascular disease [28,29,30]. The various anti-inflammatory properties of statins have stimulated numerous studies, prompting extensive investigations into their efficacy in a wide range of diseases. But could there be a potential positive effect of statins on individuals with allergic asthma [29,30,31,32,33,34]? Current therapies for asthma management primarily include bronchodilators, corticosteroids, leukotriene receptor antagonists and biologic therapies. While bronchodilators mainly provide symptom relief, corticosteroids, leukotriene receptor antagonists and biological therapies also target key aspects of the underlying pathogenesis of the disease [35,36]. In the last decade, increasing evidence suggests that statins, inhibitors of HMG-CoA reductase, offer greater benefits than their cholesterol-lowering effects. These additional pleiotropic-effects, including immunomodulatory, anti-inflammatory and antioxidant properties, may provide additional benefits [29,30,32]. Exploiting these diverse statin-induced protective effects may provide therapeutic opportunities for addressing chronic lung diseases that are characterized by inflammation and oxidative stress, such as allergic asthma [32,37]. Statins, recognized for their effectiveness in treating cardiovascular conditions like atherosclerosis, may also demonstrate the ability to relax vascular smooth muscle. A recent study showed that simvastatin induces vascular relaxation in rat thoracic aorta [31].

Rosuvastatin belongs to a newer generation of statins, with multiple effects. It has a strong affinity for the active site of HMG-CoA reductase, having a higher potency to inhibit enzyme activity and cholesterol synthesis in vitro compared to other statins [37]. In particular, rosuvastatin is recognized for its more robust pleiotropic effects and greater efficacy in lowering LDL-cholesterol than other statins at comparable dosages and with fewer adverse effects [29,33,38].

The administration of rosuvastatin in asthmatic patients for eight weeks led to a reduction in the percentage of sputum eosinophilia, as well as in the levels of IL-6 and TNF-alpha [39].

There are studies describing the effect produced by an acute in vitro application of rosuvastatin on the aortic segments of rats with a standard or cafeteria-style diet [39]. However, to our knowledge, to date, no study has yet examined the effects of acute in vitro application of rosuvastatin on aortic segments of rats with vascular dysfunction induced by HFrDs, nor the interrelationship between HFrDs and rosuvastatin administration throughout the diet [40].

The present study aimed to investigate the potential interaction between high fructose diet (HFrD)-induced vascular dysfunction and allergic asthma, while also evaluating the therapeutic effect of rosuvastatin. This effect is likely attributed to rosuvastatin’s anti-inflammatory and pleiotropic properties, which may counteract the detrimental vascular remodeling caused by chronic inflammation and oxidative stress associated with these conditions. The interaction between vascular dysfunction and allergic asthma was explored through comprehensive assessments, including vascular reactivity measurements using an isolated organ bath myography system, serial blood analyses and histopathological examination of pulmonary and aortic tissues. The study utilized rat models of ovalbumin-induced allergic asthma, HFrD-induced vascular dysfunction and a combination of both conditions.

## 2. Materials and Methods

### 2.1. Drugs

Potassium chloride (KCl, Sigma-Aldrich, St. Louis, MO, USA), N^G^-nitro-L-arginine methyl ester (L-NAME, Sigma-Aldrich, St. Louis, MO, USA), acetylcholine chloride (Ach, Sigma-Aldrich, St. Louis, MO, USA), phenylephrine (Sigma-Aldrich, St. Louis, MO, USA), rosuvastatin (Biozyme, Cluj-Napoca, Romania), ovalbumin (OVA, grade V, Sigma-Aldrich, St. Louis, MO, USA), aluminum hydroxide (Al(OH)_3_, Sigma-Aldrich, St. Louis, MO, USA) and D-(-)-fructose (>99% purity, Sigma-Aldrich, St. Louis, MO, USA) were used. L-NAME, phenylephrine, acetylcholine chloride and potassium chloride were dissolved in distilled water. Rosuvastatin solutions were dissolved in dimethyl sulfoxide (DMSO—Sigma-Aldrich, St. Louis, MO, USA). Fresh solutions were made for each experiment.

### 2.2. Animals

Forty-eight male and female Sprague-Dawley rats, weighing 310.63 ± 15.36 g and aged 10–12 weeks, were obtained from INCDMI “Cantacuzino” (Bucharest, Romania). The animals are housed under standard laboratory conditions in cages (3 rats in each) in a special temperature-controlled room (22 ± 2 °C and 50% ± 5% humidity) with a 12:12 h light/dark cycle (lights on between 08:00 and 20:00), with ad libitum access to food and fresh water. The animals were allowed to acclimatize to the animal house conditions for two weeks prior to the start of the research. Prior permission for animal experimentation was obtained from the Bioethics Committee of the King Michael I University of Life Sciences (Timișoara, Romania), approval no. 276/17.11.2023 and also from the Ethics Committee of the Victor Babeș University of Medicine and Pharmacy (Timișoara, Romania), approval no. 40/20.12.2023. The studies were conducted in accordance with the legislation regulating animal studies, respecting the ethical guidelines for animal experimentation.

### 2.3. Experimental Design

**Figure 1:**
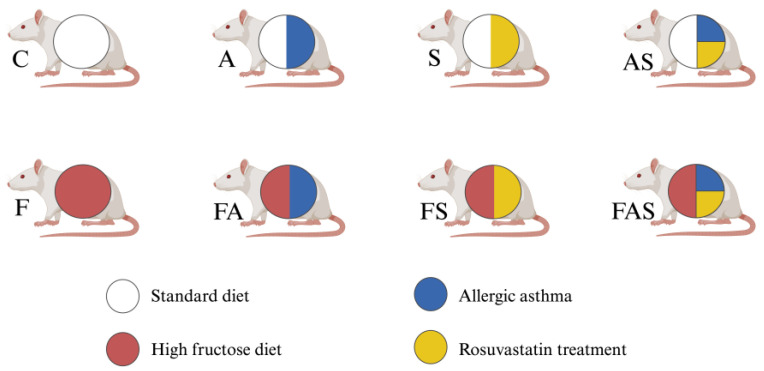
The conditions and treatments for each group (*n* = 6/group). Each letter represents a specific condition assigned to a group: C—control; A—allergic asthma; S—rosuvastatin treatment (statin); AS—allergic asthma and rosuvastatin treatment; F—high fructose diet; FA—high-fructose diet and allergic asthma; FS—high-fructose diet and rosuvastatin treatment; FAS—high-fructose diet, allergic asthma and rosuvastatin treatment. Standard diet consisted of combined rat and mouse chow. High fructose diet composed of the standard diet combined with drinking water fortified with 30% fructose. Treatment with rosuvastatin administered by gavage (40 mg/kgbw/day) throughout the study, for 12 weeks and additionally via intraperitoneal injections during the final 7 days prior to sacrifice. Allergic asthma was induced with 1 mg/mL ovalbumin and aluminum hydroxide solution administered intraperitoneally and intratracheally as shown in Figure 2. Created with BioRender.com.

The research animals (*n* = 48) were randomly divided into 8 groups (6 rats per group, with an equal distribution of males and females), of which 4 groups were fed a standard diet, with combined rat and mouse chow (the specifications are described in Appendix A, Table A1) produced and supplied by INCDMI “Cantacuzino” (Bucharest, Romania). The other four were fed a HFrD for a period of 12 weeks. The HFrD is composed of the standard diet combined with drinking water fortified with 30% fructose, consistent with protocols used in earlier research to induce metabolic and vascular dysfunction [41,42,43]. The conditions for each group are as shown in Figure 1.

**Figure 2:**
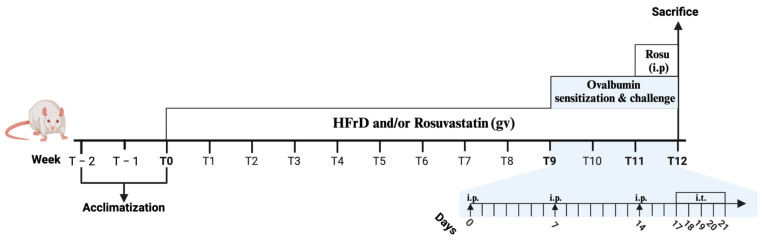
Experimental protocol. The experimental design included a 2 week acclimatization period prior to the initiation of the group specific conditions (from week T − 2 to T0). At T0, the high-fructose diet and rosuvastatin treatment (administered via gavage) were introduced for the designated groups. Ovalbumin sensitization and challenges, intended to induce allergic asthma, were carried out during the final 3 weeks of the study (T9 to T12), as illustrated above. Sensitization was performed using ovalbumin adsorbed to aluminum hydroxide in saline, followed by an intratracheal challenge to provoke the allergic response. Rosuvastatin (Rosu) was also administered intraperitoneally during the last seven days prior to sacrifice to ensure that its acute pharmacological effects were maximized during the critical period of tissue collection and analysis. This approach complements the long-term administration via gavage, allowing for both the sustained and immediate effects to be evaluated. Abbreviations: gv, gavage; i.p., intraperitoneal injection; i.t., intratracheal instillations. Created with BioRender.com.

Specific groups (A, FA, AS and FAS groups) underwent a two-phase protocol to induce allergic asthma in rats from week 9 to week 12 (T9 to T12), as illustrated in Figure 2.

During the sensitization phase, rats received intraperitoneal (i.p.) injections with 100 µg of ovalbumin (OVA) adsorbed to 4 mg of aluminum hydroxide in saline on days 0, 7 and 14, starting 3 weeks preceding euthanasia (week T9). A fresh suspension containing 2 mg/mL of ovalbumin and 80 mg/mL of aluminum hydroxide in sterile saline (0.9% NaCl) was prepared prior to administration. To ensure the proper adsorption of ovalbumin to aluminum hydroxide, the mixture was gently homogenized on a rotator for 2 h at 4 °C. The resulting white suspension, prone to settling, was brought to room temperature (18–25 °C) on the rotator before use and re-homogenized by inverting the tube or syringe 3 to 4 times immediately prior to administration. Under general anesthesia, each rat received 1 mL of the suspension via i.p. injection using a 1 mL syringe and a 25G needle.

Following sensitization, the challenge phase was performed on days 17, 18, 19, 20 and 21 in the final week before sacrifice (week T11). Under general anesthesia induced by 5% isoflurane and oxygen (with maintenance at 2% isoflurane), sensitized rats received intratracheal (i.t.) instillations of 20 µg of OVA in saline to induce asthma symptoms. In contrast, non-asthmatic groups (C, F, S and FS) underwent equivalent i.p. injections and i.t. instillations using saline (0.9% NaCl), which did not elicit allergic asthma. All procedures were conducted under general anesthesia to minimize distress. Experimental assessments were performed 18–24 h after the final i.t. challenge to evaluate the effects of OVA sensitization and challenge on allergic asthma.

Specific groups (S, FS, AS and FAS) were treated with rosuvastatin, which was administered daily throughout the study, beginning at T0, via oral gavage at a dose of 40 mg/kg body weight. To enhance the therapeutic effect and ensure consistent plasma levels during the final stage of the experiment, the rosuvastatin regimen was supplemented with intraperitoneal (i.p.) injections of the same dose (40 mg/kg body weight) during the final seven consecutive days prior to euthanasia (week T11). The experimental timeline and protocol are described in Figure 2.

### 2.4. Measurement of Anthropometric Parameters

Body weight (BW) was measured at the beginning of the study, after 6 weeks and before sacrifice, with a digital balance. For the measurement of abdominal circumference (AC), the rat was positioned in a supine position, with the limbs spaced slightly from the rest of the trunk, and the determination was made in the midline of the abdomen.

### 2.5. Analysis of Blood Parameters

**Figure 3:**
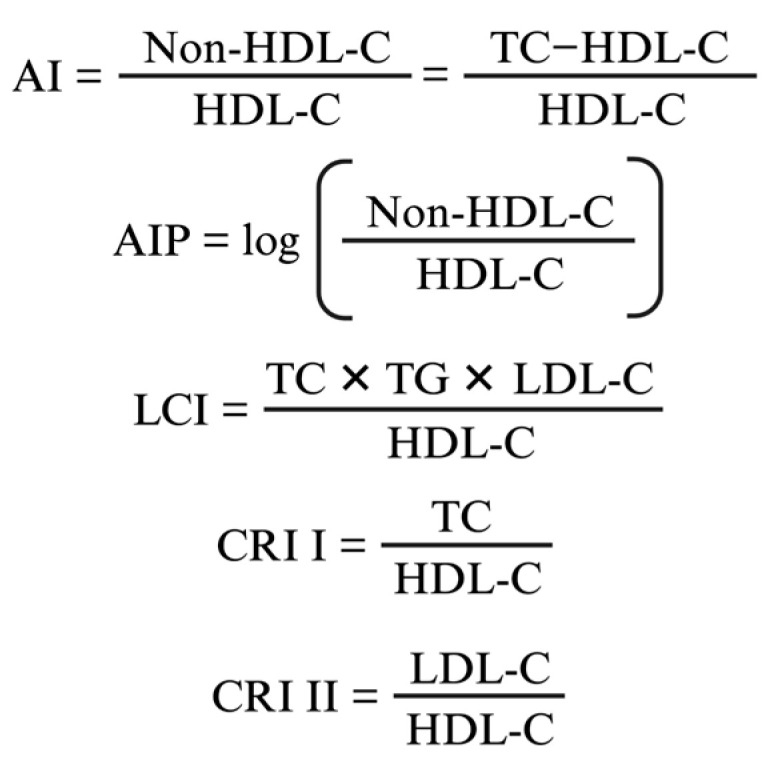
Composite lipid indices. AI—atherogenic index; AIP—atherogenic index of plasma; LCI—lipoprotein combine index; CRI I—Castelli risk index I; CRI II—Castelli risk index II; TC—total serum cholesterol; HDL-C—high-density lipoprotein-cholesterol; LDL-C—low-density lipoprotein-cholesterol; TG—serum triglycerides.

Peripheral blood was drawn from the rat’s lateral tail vein before the experiment, 6 weeks after the initiation of group-specific conditions and at the end of experiment, before the sacrifice. It was subsequently centrifuged at 3500 rpm for 5 min at 4 °C. Serum was stored at 4 °C until it was used in assays on the same day. Serum triglycerides (TG), total serum cholesterol (TC), high-density lipoprotein-cholesterol (HDL-C), low-density lipoprotein-cholesterol (LDL-C), aspartate transaminase (AST), alanine transaminase (ALT), serum glycemia (GLY), C-reactive protein (CRP), serum immunoglobulin E (IgE) were measured using commercial kits following the manufacturer’s instructions (with Beckman Coulter DxC 700 AU, Beckman Coulter Inc., Brea, CA, USA). The composite lipid parameters (atherogenic index, atherogenic index of plasma, lipoprotein combine index, Castelli risk index I, Castelli risk index II) were determined using the formulae shown in Figure 3.

### 2.6. Organ Bath Myography System Protocol

Rats were sacrificed under general anesthesia by cervical dislocations, after an overnight fasting. The descending thoracic aortas were immediately dissected and excised, placed in cold buffer, then cleaned and freed from surrounding connective tissue. The isolated aortas were cut into 4–5 mm long rings. The rings were then placed in the organ bath chamber containing 10 mL Krebs–Henseleit solution (KHS, mM: NaCl 118, KCl 4.70, MgSO_4_ 0.6, KH_2_PO_4_ 2.150, NaHCO_3_ 25, CaCl_2_ 1.69, Glucose 11), which were maintained at pH 7.4, thermoregulated at 37 °C and aerated (95% O_4_ and 5% CO_2_), as shown in Figure S1.

Changes in isometric tension of aortic rings were recorded using a four-channel force-displacement transducer (EXP-SG-4, Serial No.: 1300363, Experimetria Ltd., Budapest, Hungary) connected through amplifiers (SOFT-08-32 A1121015, Serial No.: 1300370, Experimetria Ltd., Budapest, Hungary) to the integrated organ bath system (ISO-08, Serial No.: 1300360, Experimetria Ltd., Budapest, Hungary) and displayed using the SPEL Advanced IsoSys v3.97 software (Experimetria Ltd., Budapest, Hungary).

Each aortic ring was mounted onto two stainless steel hooks, with the upper one attached to the force transducer connected to the data acquisition system. The conditions required in the organ bath chambers were precisely controlled using a heater circulating pump (Radnoti 170051G, ADInstruments Inc., Colorado Springs, CO, USA) and a carbogen gas tank (5% CO_2_ with 95% O_2_, Linde Gaz, Timisoara, Romania). The optimal tension was selected from the preliminary trials that had the greatest response to phenylephrine (10^−6^ M). The aortic rings were given approximately 2.0 g of initial tension and were then allowed to stabilize for 1 h.

To confirm the viability of the aortic rings and the integrity of the endothelium, two preliminary tests were performed. First, the viability of the aortic segments was assessed by inducing two isometric contractions using KCl (70 mM) to ensure smooth muscle responsiveness. This was followed by a contraction induced by phenylephrine (10^−^⁶ M) to further validate smooth muscle functionality. Endothelial integrity was evaluated by administering acetylcholine (Ach, 10^−5^ M). Aortic rings were considered to have intact endothelium if they achieved at least 30% relaxation of the phenylephrine-induced contraction upon exposure to Ach. This threshold aligns with the established criteria from similar studies using the organ bath system to evaluate endothelial function in rat aortic rings [44,45,46]. Then, the rings were rinsed three times with KHS to restore tension to precontraction levels.

Afterwards, two sets of experiments were conducted to assess the effects of rosuvastatin and acetylcholine-induced vasorelaxation on phenylephrine-precontracted rat aortic rings from each group.

Foe the first set of experiments, 60 min after restoration of basal tension, KCl 70 mM was added to check the viability of the aortas. Then, phenylephrine 10^−6^ M was added to induce contraction in endothelium-intact aortic rings from each rat. After the contraction plateau was reached, rosuvastatin (10^−9^→10^−4^ M) was cumulatively added to the organ bath at 5 min intervals. The tension changes were reported as a percentage of the maximal phenylephrine-induced contraction in these aortic rings. The same protocol was followed to assess the relaxation induced by acetylcholine (10^−9^→10^−4^ M) on phenylephrine pre-contracted aortic rings.

For the second set of experiments, to investigate the role of nitric oxide (NO) in the relaxation response, the effects of rosuvastatin or acetylcholine were studied in the presence of nitric oxide synthase inhibitor L-NAME (10^−5^ M). Aortic rings were pre-incubated for 30 min with 10^−5^ M L-NAME, an inhibitor of vasorelaxation, followed by phenylephrine pre-contraction. After reaching the contraction plateau, increasing concentrations of rosuvastatin or acetylcholine were added at 5-min intervals. The vasorelaxation responses were measured to assess the involvement of nitric oxide in mediating the observed effects.

### 2.7. Histomorphometric Analysis of Aortic and Pulmonary Tissues

Aortic and pulmonary samples were collected following the sacrifice at the end of the experiment. After fixation in formaldehyde, the tissues were paraffin-embedded, sectioned into 4–5 μm slices and stained with hematoxylin and eosin (H&E). The cross-sectional areas of the abdominal aorta and lungs were evaluated by a pathologist in a single-blind manner. Randomly selected stained sections of aortic and lung tissues were photographed using the Invitrogen EVOS™ FL Auto 2 Imaging System (Thermo Fisher Scientific, Bothell, WA, USA).

For the lung sections, we evaluated the thickness of the segmental bronchial walls and assessed the degree of inflammatory infiltrate, which was graded on a scale from 0 to 3 as follows: 0—no signs of inflammation, 1—slight inflammation, 2—moderate inflammation, 3—severe inflammation. For the bronchial wall thickness, measurements taken included both the mucosa and smooth muscle tissue. To ensure accuracy, three separate bronchiolar structures were evaluated at three distinct locations, and the values were averaged. For the inflammatory infiltrate, the bronchiolar structures present on the slide were evaluated globally.

Morphometric evaluations of the abdominal aortas included measurements of total wall thickness (μm). The average wall thickness measurements for each artery were calculated by averaging the individual measurements.

### 2.8. Statistical Analysis

In all experiments, ‘n’ corresponds to the number of animals from which aortic rings were extracted (6 in each instance) and the results were presented as mean ± standard deviation. The maximal smooth muscle contraction levels induced by phenylephrine were considered as 100%, and the mean percentages of muscle tone values post-drug administration were utilized in the statistical analysis. Multiple comparisons were conducted using the one-way analysis of variance (ANOVA) test, and differences between groups were determined by the Bonferroni post hoc test. Additionally, the effects of inhibitors on rosuvastatin- or acetylcholine-induced relaxation of aortic rings (across all rat groups) were evaluated using repeated measures of two-way analysis of variance (RM-ANOVA), followed by post hoc tests as deemed appropriate. Statistical analysis for blood and anthropometric parameters was performed using one-way or two-way ANOVA followed by Tukey’s or Sidak’s multiple comparison test. The statistical program used was GraphPad Prism version 8.3.1 (GraphPad Software, Boston, MA, USA). Statistical significance was defined as *p* < 0.05.

## 3. Results

Values of the anthropometric parameters (including BW, AC), TC, HDL-C, LDL-C, TG, AST, ALT, GLY, IgE and CRP were assessed before and after the 12 week period for all Sprague-Dawley rats. These measurements took into account the type of diet (standard diet or high-fructose diet) and treatment conditions (no treatment, rosuvastatin treatment and/or ovalbumin-induced allergic asthma).

### 3.1. Anthropometric Parameters

**Figure 4:**
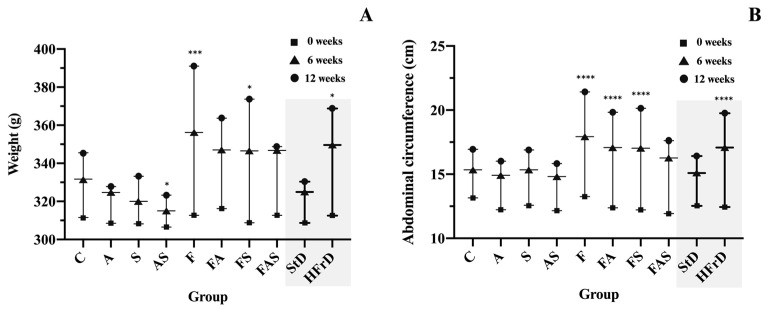
(**A**) Body weight and(**B**) abdominal circumference evolution at 0, 6 and 12 weeks from the initiation of group-specific conditions. The treatments and conditions for each group (*n* = 6/group): C—control; A—allergic asthma; S—rosuvastatin treatment; AS—allergic asthma and rosuvastatin treatment; F—high-fructose diet; FA—high-fructose diet and allergic asthma; FS—high-fructose diet and rosuvastatin treatment; FAS—high-fructose diet, allergic asthma and rosuvastatin treatment; StD—all standard diet groups; HFrD—all high fructose diet groups. All protocols are presented above in Figure 2. * *p* < 0.05, *** *p* < 0.001, **** *p* < 0.0001 versus control. The shadowed area in the figure is intended to highlight the comparison between all the Standard Diet (StD) groups versus the High-Fructose Diet (HFrD) groups.

The initial BW showed no statistically significant differences across the experimental groups (310.63 g ± 15.36 g, *p* > 0.05). Alternatively, rats on a HFrD for 12 weeks exhibited a notable increase in body weight compared to those on a standard diet (391.00 g ± 11.98 g vs. 345.50 g ± 16.86 g, *p* < 0.001). However, the groups with induced allergic asthma exhibited a stabilization in BW gain compared to the control group following the initial challenge with ovalbumin until sacrifice (Figure 4).

Rats in the HFrD groups exhibited a significant increase in AC compared to the standard diet groups (at 6 weeks, an increase of 4.63 ± 0.76 cm in HFrD groups vs. 2.58 ± 0.77 cm in standard diet groups, *p* < 0.0001 and at 12 weeks an increase of 7.31 ± 1.37 cm in HFrD groups vs. 3.90 ± 0.77 cm in standard diet groups, *p* < 0.0001). Similar to the progression of BW, the AC in allergic asthma groups showed a plateau in the rate of increase following the sensitization, persisting until sacrifice.

### 3.2. The Impact on Serum Biomarkers

The further implications of a multi-factor regimen, including OVA sensitization and challenge and the diet scheme mentioned above, were examined through a series of serum parameters.

**Figure 5:**
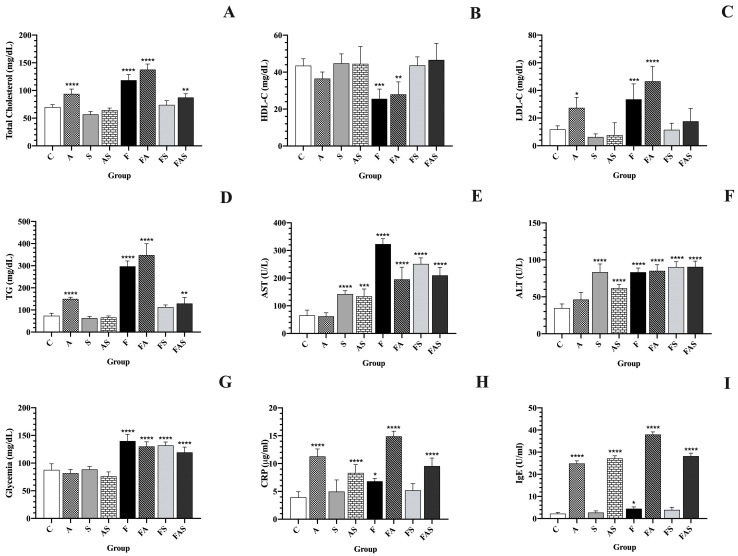
Blood analysis parameters of rats after a 12 week regimen. Data are shown as mean ± SD. Statistical analysis was performed using one-way analysis of variance (ANOVA) followed by Tukey’s multiple comparison test. (**A**) Total serum cholesterol (mg/dL); (**B**) HDL-C—high-density lipoprotein-cholesterol (mg/dL); (**C**) LDL-C—low-density lipoprotein-cholesterol (mg/dL); (**D**) TG—serum triglycerides (mg/dL); (**E**) AST—aspartate transaminase (U/L); (**F**) ALT—alanine transaminase (U/L); (**G**) glycemia (mg/dL); (**H**) CRP—C-reactive protein (µg/mL); (**I**) IgE—immunoglobulin E (U/mL). The treatments and conditions for each group (*n* = 6/group): C—control; A—allergic asthma; S—rosuvastatin treatment; AS—allergic asthma and rosuvastatin treatment; F—high-fructose diet; FA—high-fructose diet and allergic asthma; FS—high-fructose diet and rosuvastatin treatment; FAS—high-fructose diet, allergic asthma and rosuvastatin treatment. * *p* < 0.05, ** *p* < 0.01, *** *p* < 0.001, **** *p* < 0.0001 compared to controls.

At the outset of the study, no statistically significant differences were observed in the serum markers across the groups. Figure 5 displays the biochemical parameters evaluated after the 12 week regimen.

Lipid profiles, including TC, LDL-C, TG were significantly elevated in the HFrD groups without statin treatment (F, FA) compared to controls (*p* < 0.0001). HDL-C was significantly lower in these groups (*p* < 0.05).

Sensitization to OVA also increased TC by 33.82%, LDL-C by 177.08% and TG by 58.31% and decreased in HDL-C by 16.05% compared to control values.

Rosuvastatin treatment demonstrated amelioration of hypercholesterolemia induced by a high-fructose diet, effectively reducing TC even in the presence of allergic asthma (FS: 74.0 ± 6.9 mg/dL vs. F: 137.7 ± 9.2 mg/dL, *p* < 0.0001; FAS: 87.4 ± 6.1 mg/dL vs. FA: 118.6 ± 9.3 mg/dL, *p* < 0.0001. Moreover, in the high-fructose diet, allergic asthma and rosuvastatin treatment group (FAS), the serum level of LDL-C was reduced compared to that in the corresponding non-treated group (FA).

The serum level of TG in the statin-treated groups (AS, FS and FAS) was also significantly decreased compared to that in the corresponding non-treated groups (A, F and FA). These findings are illustrated in Figure 5.

Additionally, rats consuming water fortified with 30% fructose for 12 weeks exhibited significantly elevated glycemic values (143.27 ± 21.65 mg/dL) compared to those on a standard diet (88.96 ± 8.18 mg/dL, *p* < 0.0001).

The current findings demonstrate that sensitization and challenges with OVA significantly elevated CRP levels compared to the control group (A: 978.3 ± 96.8 μg/mL vs. C: 413.2 ± 117.7 μg/mL, *p* < 0.001), suggesting that CRP could serve as a marker for systemic inflammation in allergic asthma. Compared to the standard diet groups under the same sensitization protocol, rats on the HFrD exhibited a 9.10% increase in serum CRP levels (FA vs. A). Rosuvastatin treatment, known for its pleiotropic effects, including lipid-lowering and anti-inflammatory properties, significantly reduced CRP levels by 34.9% in FS vs. F, by 27.62% in FAS vs. FA and by 21.67% in AS vs. A. However, no significant effect was observed in S vs. C.

Allergen challenge elevated IgE serum levels, resulting in a 4.5-fold increase in the A group compared to the control group. Additionally, rats with HFrD-induced vascular dysfunction exhibited significantly higher total serum IgE levels compared to the standard diet group under the same sensitization protocol (FA vs. A, *p* < 0.05). Rosuvastatin treatment significantly reduced IgE levels by 31.58% in FS vs. F and by 25.45% in FAS vs. FA, although it had no effect in S vs. C.

**Figure 6:**
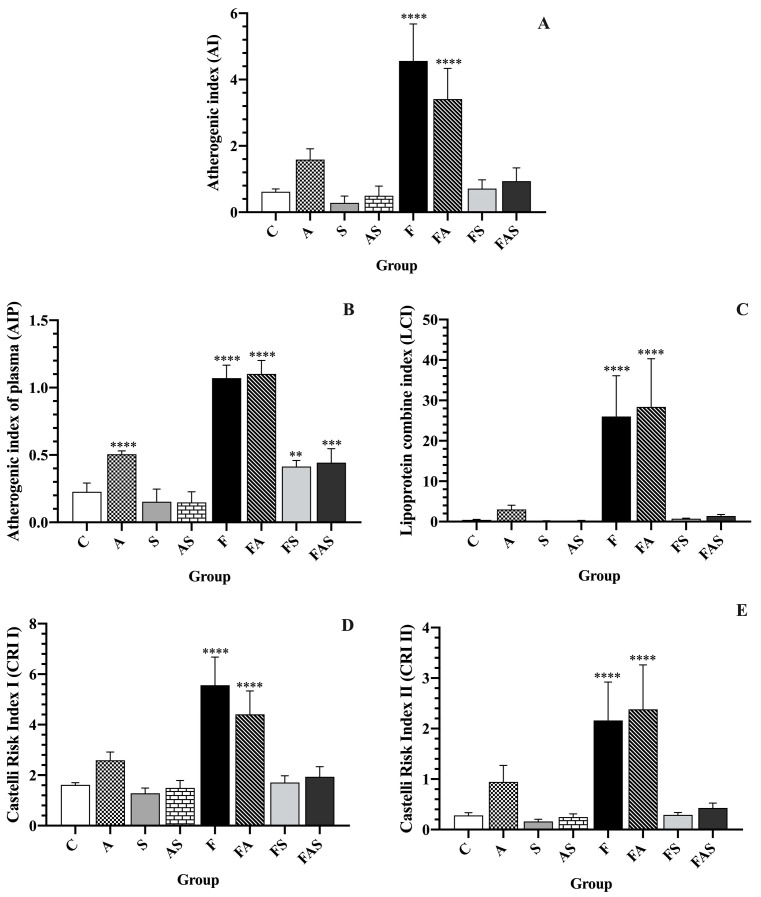
Composite lipid indices. Data are shown as mean ± SD (*n* = 6 in each group). ** *p* < 0.005, *** *p* < 0.001, **** *p* < 0.0001 versus control. Statistical analysis was performed using one-way analysis of variance (ANOVA) followed by Tukey’s multiple comparison test. The treatments and conditions for each group (*n* = 6/group): C—control; A—allergic asthma; S—rosuvastatin treatment; AS—allergic asthma and rosuvastatin treatment; F—high-fructose diet; FA—high-fructose diet and allergic asthma; FS—high-fructose diet and rosuvastatin treatment; FAS—high-fructose diet, allergic asthma and rosuvastatin treatment. (**A**) AI—atherogenic index = (TC − HDL-C)/HDL-C; (**B**) AIP—atherogenic index of plasma = log (TG/HDL-C); (**C**) LCI—lipoprotein combine index = TC × TG × LDL-C/HDL-C; (**D**) CRI I—Castelli risk index I = TC/HDL-C; (**E**) CRI II: Castelli risk index II = LDL-C/HDL-C. TC—total serum cholesterol; HDL-C—high-density lipoprotein-cholesterol; LDL-C—low-density lipoprotein-cholesterol; TG—serum triglycerides.

Figure 6 shows the composite lipid indices (AI, AIP, LCI, CR I and CR II) levels of rats with HFrD-induced vascular dysfunction, rosuvastatin treatment and/or allergic asthma. The results demonstrated that a HFrD significantly elevates all composite lipid indices levels compared to the control group (*p* < 0.0001).

Rosuvastatin treatment significantly improved the composite lipid indices, including AI, AIP, LCI, CRI and CRII, as follows:

Regarding AI and AIP, statin-treated groups showed significant improvement compared to non-statin-treated corresponding groups: AI: F (4.56 ± 1.11) vs. FS (0.71 ± 0.26), *p* < 0.0001; A (1.59 ± 0.33) vs. AS (0.50 ± 0.29), *p* < 0.05; FA (3.41 ± 0.92) vs. FAS (0.94 ± 0.40), *p* < 0.0001. AIP: F (1.07 ± 0.10) vs. FS (0.41 ± 0.05), *p* < 0.0001; A (0.51 ± 0.02) vs. AS (0.15 ± 0.08), *p* < 0.0001; FA (1.10 ± 0.10) vs. FAS (0.44 ± 0.10), *p* < 0.0001.

On the other hand, regarding LCI and CRII, there was no significant difference between: A vs. AS: LCI: A (3.03 ± 1.05) vs. AS (0.28 ± 0.05), *p* = 0.97, CRII: A (0.95 ± 0.33) vs. AS (0.25 ± 0.06), *p* = 0.12. However, rosuvastatin treatment significantly improved F and FA: F − LCI (26.03 ± 10.05) and CRII (2.16 ± 0.76) vs. FS − LCI (0.71 ± 0.15) and CRII (1.71 ± 0.26), *p* < 0.0001, FA − LCI (28.38 ± 11.92) and CRII (4.41 ± 0.92) vs. FAS − LCI (1.41 ± 0.37) and CRII (1.94 ± 0.40), *p* < 0.0001.

Concerning CRI, we observed significant improvements in statin-treated groups: F (5.56 ± 1.11) vs. FS (1.71 ± 0.26), *p* < 0.0001; A (2.59 ± 0.33) vs. AS (1.50 ± 0.29), *p* < 0.001; FA (4.41 ± 0.92) vs. FAS (1.94 ± 0.40), *p* < 0.0001.

These findings emphasize the significant benefits of rosuvastatin treatment in improving the lipid profile, particularly across the composite lipid indices.

### 3.3. Organ Bath Vascular Reactivity

#### 3.3.1. Evaluation of Aortic Reactivity and Vascular Relaxation Induced by Rosuvastatin and Acetylcholine

**Figure 7:**
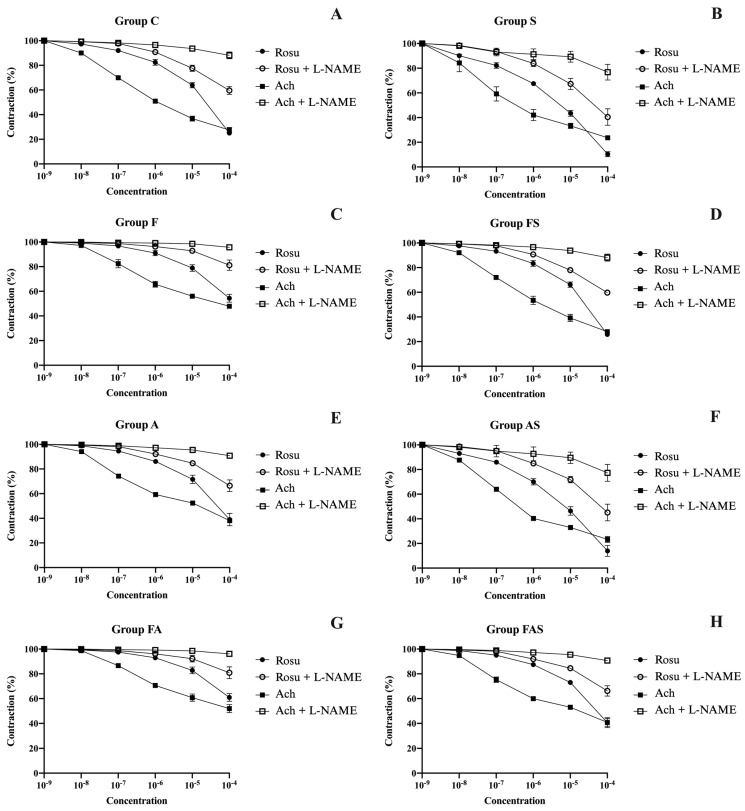
Organ bath aortic reactivity. Panels (**A**–**H**) illustrate the mean relaxation responses of aortic rings for each group, starting from 100% phenylephrine-induced contraction. Data are shown as mean ± SD (*n* = 6 in each group). Relaxation was induced using increasing concentrations (10^−9^ M to 10^−4^ M) of either rosuvastatin or acetylcholine, with or without preincubation with L-NAME. Each panel represents the mean changes in aortic tension following the addition of: Rosu (rosuvastatin), Ach (acetylcholine), Rosu + L-NAME (preincubation with L-NAME followed by rosuvastatin relaxation after phenylephrine precontraction), or Ach + L-NAME (preincubation with L-NAME followed by acetylcholine relaxation after phenylephrine precontraction). The treatments and conditions for each group (*n* = 6/group): C—control; A—allergic asthma; S—rosuvastatin treatment; AS—allergic asthma and rosuvastatin treatment; F—high-fructose diet; FA—high-fructose diet and allergic asthma; FS—high-fructose diet and rosuvastatin treatment; FAS—high-fructose diet, allergic asthma and rosuvastatin treatment. Rosu—rosuvastatin; L-NAME—N^G^-nitro-L-arginine methyl ester; Ach—acetylcholine. All statistical analyses supporting these findings are detailed and discussed in Section 3.3.

To investigate the relaxation response of precontracted aortic rings, cumulative concentrations of rosuvastatin, ranging from 10^−9^ to 10^−4^ M, were sequentially introduced into the tissue bath. The derived degree of relaxation, in comparison with the maximum contraction induced by phenylephrine for each group, as illustrated in Figure 7, is expressed by a percentage, as follows (Rosuvastatin and Ach, %): (a) 25.07 ± 1.27% and 27.55 ± 2.07% for the C group; (b) 54.33 ± 3.15% (*p* < 0.0001) and 47.86 ± 1.70% (*p* < 0.0001) for the F group; (c) 25.79 ± 1.89% (*p* > 0.05) and 28.01 ± 1.96% (*p* > 0.05) for the A group; (d) 10.35 ± 2.03% (*p* < 0.0001) and 23.62 ± 1.61% (*p* < 0.0001) for the S group; (e) 60.98 ± 3.12% (*p* < 0.0001) and 51.93 ± 3.27% (*p* < 0.0001) for the FA group; (f) 38.95 ± 5.03% (*p* < 0.0001) and 38.1 ± 1.95% (*p* < 0.0001) for the FS group; (g) 13.96 ± 4.39% (*p* < 0.0001) and 23.32 ± 2.41% (*p* < 0.0001) for the AS group; (h) 40.61 ± 4.01% (*p* < 0.0001) and 40.8 ± 3.11% (*p* < 0.0001) for the FAS group.

The strongest vasorelaxation was recorded with rosuvastatin in the S group (*p* < 0.0001), confirming its potent vasodilatory effect in healthy rats.

For both types of mediators, acetylcholine and rosuvastatin, the AS group demonstrated a significant relaxant response (*p* < 0.0001), highlighting the efficacy of rosuvastatin in enhancing vascular function under asthma-induced inflammatory conditions. In contrast, the A group showed no significant difference compared to the C group (*p* > 0.05), indicating that asthma alone does not substantially impair vascular reactivity in the absence of additional factors. Similarly, both the FS and FAS groups displayed comparable responses (*p* > 0.05), with reduced relaxation compared to the C group (*p* < 0.0001), highlighting the detrimental impact of a high-fructose diet on vasorelaxation.

When analyzing the effects of rosuvastatin-induced relaxation across groups with and without a high-fructose diet (C vs. F; A vs. FA; S vs. FS; AS vs. FAS), we observed a marked and highly significant impairment of vascular function in the HFrD groups compared to their non-HFrD counterparts (*p* < 0.0001 for all comparisons). These findings emphasize the profound detrimental impact of a HFrD on vascular reactivity across the studied conditions. Notably, the FA group exhibited the weakest response, demonstrating the compounded negative impact of both the high-fructose diet and allergic asthma on endothelial function.

Regarding asthma’s influence on relaxation, comparisons between non-asthmatic and asthmatic groups (C vs. A; F vs. FA; S vs. AS; FS vs. FAS) showed a significant reduction in vasorelaxation for the C vs. A and FS vs. FAS comparisons (*p* < 0.0001). However, the impact of asthma was less pronounced when comparing S vs. AS (*p* < 0.05). Additionally, a weaker vascular relaxation response was evident in the FA group compared to the F group (*p* < 0.001). These results indicate that, while asthma negatively influences vascular reactivity, its overall impact is less severe than the pronounced impact of the high fructose diet, which remains the predominant factor contributing to vascular dysfunction.

Finally, comparisons between statin-treated groups and their non-statin counterparts (C vs. S; A vs. AS; F vs. FS; FA vs. FAS), revealed that rosuvastatin treatment consistently resulted in a significant improvement in vascular relaxation (*p* < 0.0001 across all comparisons). These findings highlight rosuvastatin’s potent vasodilatory, pleiotropic and protective effects, emphasizing its therapeutic potential in alleviating vascular dysfunction associated with inflammatory and metabolic conditions.

#### 3.3.2. Assessment of Rosuvastatin and Acetylcholine-Induced Aortic Relaxation Following L-NAME Incubation

To further explore the mechanism underlying the observed vasorelaxant effects, a second set of experiments were conducted.

Aortic rings were pre-incubated with the nitric oxide synthase inhibitor L-NAME (10^−5^ M) for 30 min, followed by the cumulative addition of rosuvastatin or acetylcholine after phenylephrine-induced contraction. The presence of L-NAME significantly reduced the relaxation response in all groups, confirming the pivotal role of nitric oxide (NO) in mediating vasorelaxation. Despite the NO inhibition, the S and AS groups still exhibited the highest relaxation (with rosuvastatin: S = 40.47 ± 6.68%, *p* < 0.0001; AS = 45.14 ± 6.74%, *p* < 0.0001; with acetylcholine: S = 76.81 ± 6.39%, *p* < 0.0001; AS = 77.33 ± 6.84%, *p* < 0.0001). The F and FA groups again showed the weakest relaxation (Rosuvastatin: F = 81.21 ± 4.21%, *p* < 0.0001; FA = 80.84 ± 4.8%, *p* < 0.0001; Ach: F = 95.72 ± 1.05%, *p* < 0.01; FA = 96.09 ± 1.14%, *p* < 0.01), indicating that metabolic and inflammatory stress, induced by a high-fructose diet and asthma, severely impairs NO-mediated vasodilation. No significant differences were observed between the A, FS, FAS and C groups for the rosuvastatin relaxation (*p* > 0.05). Moreover, the groups FAS, FS, FA and F exhibited no statistical differences (*p* > 0.05) regarding L-NAME-impaired relaxation upon the addition of acetylcholine. These findings emphasize that acetylcholine-induced relaxation is more dependent on nitric oxide-mediated mechanisms, in contrast to rosuvastatin, which seems to engage additional complementary pathways contributing to vasodilation. Further investigation is essential and would make a significant advancement by elucidating these mechanisms within this research domain.

Figure 7 illustrates the aortic reactivity of all experimental groups as measured using the organ bath myography system.

### 3.4. Histomorphometric Analysis of Pulmonary and Vascular Tissues

**Figure 8:**
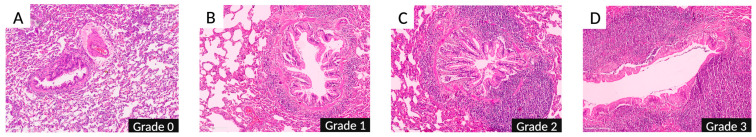
Grading of the inflammatory infiltrate in the pulmonary tissue. (**A**) Grade 0: no signs of inflammation; (**B**) Grade 1: slight inflammation; (**C**) Grade 2: moderate inflammation; (**D**) Grade 3: severe inflammation. View of the pulmonary fragments at 20× magnification. Scale bar: 200 μm.

**Figure 9:**
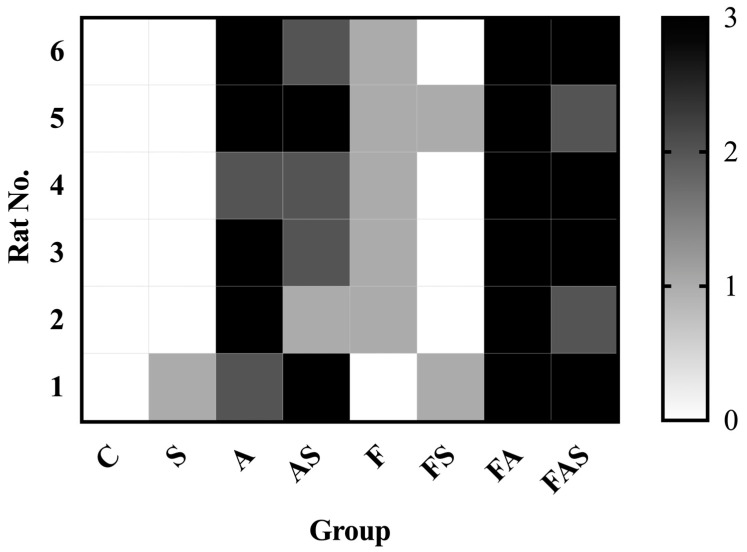
Grading of the inflammatory infiltrate in the pulmonary tissue. 0: No signs of inflammation; 1: Slight inflammation; 2: Moderate inflammation; 3: Severe inflammation. The treatments and conditions for each group (*n* = 6/group): C—control; A—allergic asthma; S—rosuvastatin treatment; AS—allergic asthma and rosuvastatin treatment; F—high-fructose diet; FA—high-fructose diet and allergic asthma; FS—high-fructose diet and rosuvastatin treatment; FAS—high-fructose diet, allergic asthma and rosuvastatin treatment.

**Figure 10:**
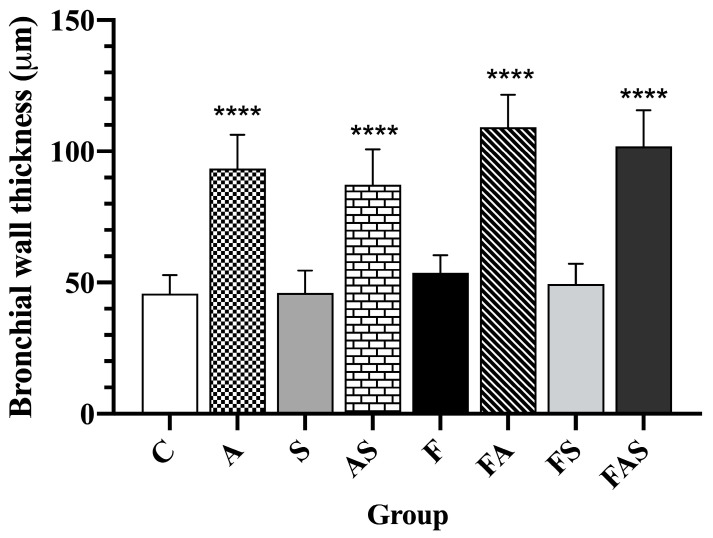
Wall thickness of segmental bronchial tissue. Data are shown as mean ± SD (*n* = 6 in each group, with three measurements per subject). **** *p* < 0.0001. Statistical analysis was performed using one-way analysis of variance (ANOVA) followed by Tukey’s multiple comparison test. The treatments and conditions for each group: C—control; A—allergic asthma; S—rosuvastatin treatment; AS—allergic asthma and rosuvastatin treatment; F—high-fructose diet; FA—high-fructose diet and allergic asthma; FS—high-fructose diet and rosuvastatin treatment; FAS—high-fructose diet, allergic asthma and rosuvastatin treatment.

Histological examination of H&E-stained lung tissues revealed the characteristic microscopic features of asthma in all groups sensitized and challenged with ovalbumin (Figure 8, Figure 9 and Figure 10). The inflammatory infiltrate in the allergic asthma group (A) ranged between grades 2 (moderate) and 3 (severe), whereas the C and S groups showed grade 0 (no inflammation). Interestingly, the HFrD group (F) exhibited a mild grade of inflammation of pulmonary tissues (grade 1). The association between a HFrD and ovalbumin sensitization and challenge (FA) demonstrated a progression of inflammation, with all examined samples displaying grade 3. In the allergic asthma group treated with rosuvastatin (AS), a reduction in inflammatory cells was noted compared to allergic asthma group (A).

An increase in mean bronchial wall thickness was observed in the allergic asthma groups compared to controls (Figure 10). Additionally, the combination of a HFrD with allergic asthma (FA) further augmented bronchial wall thickness compared to allergic asthma alone (A) (C = 45.83 ± 7.03 μm vs. A = 93.44 ± 12.90 μm, *p* < 0.0001; F = 53.72 ± 6.68 μm vs. FA = 110.17 ± 12.72 μm, *p* < 0.0001, A = 93.44 ± 12.90 μm vs. FA = 110.17 ± 12.72, *p* < 0.05). The corresponding statin-treated groups showed a slight reduction in the mean wall thickness, though this was not statistically significant (A = 93.44 ± 12.90 μm vs. AS = 87.28 ± 13.49 μm; FA = 110.17 ± 12.72 μm vs. FAS = 101.89 ± 13.75 μm).

**Figure 11:**
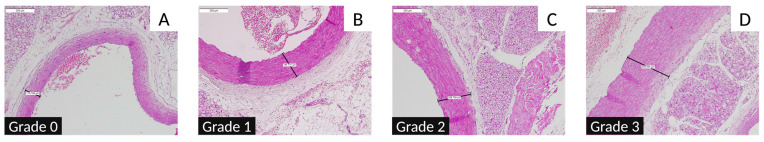
Grading the alterations of the abdominal aorta histology. (**A**) Grade 0: no thickening of the aortic wall (<140 µm), (**B**) Grade 1: slight thickening of the aortic wall (140–210 µm), (**C**) Grade 2: moderate thickening of the aortic wall (210–280 µm), (**D**) Grade 3: important thickening of the aortic wall (>280 µm).

The histological features of the abdominal aortas’ cross-sections across all experimental groups are illustrated in Figure 11.

**Figure 12:**
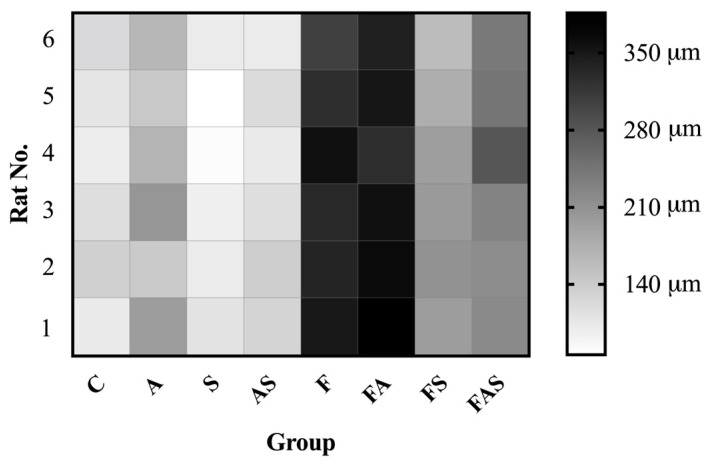
Assessment of the mean aortic wall thickness across the experimental groups. The treatments and conditions for each group (*n* = 6/group): C—control; A—allergic asthma; S—rosuvastatin treatment; AS—allergic asthma and rosuvastatin treatment; F—high-fructose diet; FA—high-fructose diet and allergic asthma; FS—high-fructose diet and rosuvastatin treatment; FAS—high-fructose diet, allergic asthma and rosuvastatin treatment.

Abdominal aortic wall thickness varied significantly among the experimental groups (Figure 12). Compared to controls, the HFrD groups exhibited a significant increase in wall thickness (C = 113.70 ± 13.11 μm; F = 333.87 ± 18.50 μm, *p* < 0.001; FA = 354.13 ± 20.52 μm, *p* < 0.001). The corresponding statin-treated groups (FS = 188.95 ± 18.53 μm, *p* < 0.001; FAS = 237.94 ± 23.32 μm, *p* < 0.001) showed a reduction in wall thickness compared to non-statin treated HFrD or allergic asthma groups (as seen in Figure 12). Allergic asthma was also associated with a significant increase in aortic wall thickness compared to the controls (C = 113.70 ± 13.11 μm; A = 169.65 ± 25.89 μm, *p* = 0.0002).

## 4. Discussion

Our major findings indicate that high-dose rosuvastatin treatment not only improves vascular reactivity in rats with high-fructose diet-induced vascular dysfunction but also effectively addresses allergic asthma, targeting both conditions concurrently. Therefore, beneficial vascular effects induced by rosuvastatin treatment may occur even under hyperglycemic conditions. Similar studies were conducted in order to establish the role of atorvastatin in the vascular function of diabetic rats [47]. In experimental studies, rats tolerate a diet with high fructose levels (even ≥60%), while in humans, gastric discomfort may develop even at lower doses of fructose (≥10%) [48].

Since high fructose intake is characterized by increased energy intake, it was expected that water supplementation with 30% fructose would correlate with higher animal body weight, corroborating our results [40,41,49]. However, the impact of high fructose consumption on body weight remains controversial. While some studies report weight gain associated with fructose intake, others observe no significant impact on BW [50,51,52,53,54].

Elevated dietary fructose levels in Sprague-Dawley rats result in an abundance of fructose converted into lipids within liver cells. This process escalates the synthesis of free fatty acids, promoting their storage within adipocytes and consequently promoting obesity. Prolonged exposure to elevated fructose levels increases circulating free fatty acids in the bloodstream. As these fatty acids are absorbed by various tissues, they contribute to obesity and eventually to the onset of insulin resistance. Prolonged consumption of a HFrD exacerbates insulin resistance and obesity, ultimately leading to the development of diabetic phenotypes. Fasting blood glucose levels in rats (after at least 8 h of fasting) above 126 mg/dL have been considered as impaired fasting glucose while levels above 135 mg/dL suggested diabetes mellitus [55,56].

Our study’s limitations include the intrinsic differences between rat models and human physiology. While rats provide valuable insights into mechanisms of disease and treatment effects, they tolerate higher levels of fructose compared to humans, which may influence the severity and nature of metabolic and vascular changes observed [14,50,51,57]. Furthermore, rats do not fully replicate the complex metabolic and physiological responses seen in humans, such as the precise regulation of fructose metabolism, variations in insulin sensitivity and other long-term impacts on human vascular health [50,52,54].

Additionally, the doses and duration of fructose intake used in our model, although effective for inducing vascular dysfunction, may not perfectly reflect human dietary patterns, where fructose consumption typically occurs at lower doses but over longer periods.

Numerous experiments have found that fructose-induced insulin resistance correlates with serum triglyceride concentration [41,52,58,59,60,61]. Elevated serum triglyceride levels have previously been associated with the development of asthma in people with obesity. As reported in our study, this would suggest that elevated triglyceride levels may be one of the neglected features that influence the development of asthma [7,8,62].

CRI I, CRI II, AIP, AI and LCI were identified as independent predictors of CAD (coronary artery disease) and they have been discussed as potential biomarkers for the estimation of CAD severity [24]. Recent studies have identified composite lipid indices, including AI, AIP and LCI as superior predictors of coronary artery disease risk compared to traditional individual lipid parameters [24,25]. Additionally, studies have shown a significant correlation between AIP and obesity, underscoring its potential as a novel lipid marker for individuals with type 2 diabetes and atherosclerosis [24,25,26,27]. In our experiments, we observed that HFrDs significantly elevated all composite lipid indices compared to the control group. Specifically, our findings indicate that ovalbumin-induced allergic asthma significantly increases the AIP. Furthermore, even with statin treatment, the adverse effects of HFrDs could not be completely attenuated, particularly in cases where a HFrD is combined with allergic asthma.

Previous studies have shown that rosuvastatin induces vascular relaxation in rat aortic rings primarily through endothelium-mediated mechanisms, consistent with the known vasoactive effects of statins [39,63,64]. Endothelial-derived factors, particularly nitric oxide and prostacyclin, are crucial for this relaxation [64]. Our study confirmed that the vasorelaxant effect of rosuvastatin was significantly reduced after a 30 min incubation with the endothelial NO synthase inhibitor, L-NAME. These results underscore the roles of the eNOS/NO pathway in rosuvastatin-induced endothelial-dependent vascular relaxation.

The responses to phenylephrine were more pronounced in aortic rings from the HFrD group compared to the control group, although rosuvastatin treatment did not modify the vascular reactivity to phenylephrine in the control group, it reduced the maximal response in the HFrD groups. These data suggest that rosuvastatin attenuates vascular hyperreactivity to phenylephrine induced by HFrDs.

Vascular dysfunction in the aorta is often associated with reduced NO bioavailability [64]. Therefore, L-NAME was used to investigate the influence of NO in vascular responsiveness in the aortas of HFrD rats treated with rosuvastatin. In the HFrD groups, the production of NO is often impaired, reducing the ability of blood vessels to properly dilate in response to stimuli. However, rosuvastatin, known for its pleiotropic effects beyond cholesterol lowering, has been shown to improve endothelial function and enhance NO bioavailability [29,39,64,65]. By improving NO signaling, rosuvastatin treatment could counteract the reduced vasodilation, thereby ameliorating vascular reactivity [39]. This indicates that rosuvastatin may help protect against endothelial dysfunction, a consequence of a high-fructose diet. When nitric oxide synthesis was inhibited (by using L-NAME), the expected improvement in vascular function from rosuvastatin was less pronounced. This suggests that the action of rosuvastatin on the blood vessels is, at least in part, dependent on the availability of nitric oxide, with results consistent with those observed in other studies [39,63,64]. Without NO, the aorta was less responsive to the statin, indicating that NO plays a critical role in mediating the vascular benefits of rosuvastatin. These findings indicate that the inhibition of nitric oxide synthesis markedly reduced the sensitivity of the aorta to rosuvastatin in both control and HFrD rats.

However, studies have shown that various concentrations of HFrDs reduce NO-mediated aortic relaxation to acetylcholine in rats compared to controls, indicating that high fructose levels are needed to impair the relaxation response to Ach. It was previously reported that in vivo administration of fructose in drinking water increased the serum total cholesterol and insulin levels, results consistent with our study [52,54,61,65,66,67]. In these studies, fructose-induced inflammation and oxidative stress in rats and exaggerated the contractile response of aorta of fructose-fed rats to both KCl and phenylephrine. These effects of fructose were attributed to different metabolic changes elicited by fructose.

Rosuvastatin, through its pleiotropic effects, enhances the expression of antioxidant enzymes that help to reduce oxidative stress by decreasing the production of reactive oxygen species (ROS). ROS contribute to endothelial dysfunction by impairing NO availability, which is crucial for maintaining vascular tone and health. By reducing ROS and boosting antioxidant defenses, rosuvastatin helps preserve NO bioavailability, restoring normal vascular function and decreasing the risk of vascular dysfunction. Furthermore, rosuvastatin activates the Nrf2 pathway, a key regulator of antioxidant gene expression, which leads to improved endothelial function and reduced vascular inflammation [68].

In our study, we observed a reduction in the vasodilatory effect of rosuvastatin/Ach administration in vitro in the allergic asthma group (A) compared to the allergic asthma in the rosuvastatin treatment group (AS). This finding may be attributed to the influence of eosinophils in allergic asthma. Eosinophils play a key role in promoting nitrosative and oxidative stress. Upon allergen exposure, they generate ROS and release eosinophil peroxidase, an enzyme that catalyzes the bromination of the amino acid tyrosine, which contribute to vascular dysfunction and may partially explain the attenuated effect of rosuvastatin/Ach in the allergic asthma group. This process is an important link between eosinophils and oxidative stress during asthma exacerbations [69]. Statins, such as rosuvastatin, have been shown to ameliorate these effects, thereby potentially alleviating oxidative and nitrosative damage in allergic asthma, outcomes consistent with those observed in our study [70,71].

Inflammatory cells, especially macrophages, monocytes and mast cells, are present in the bronchoalveolar vessel walls of asthmatic subjects, but also in atherosclerotic lesions. This may suggest that they play a crucial role in the pathogenesis of both conditions and perform comparable functions. In a recent study, we analyzed the correlation between IgE and CRP levels in rats with allergic asthma induced by ovalbumin, ragweed and/or house dust mites, combined with a HFrD (with 40% fructose in drinking water). The findings demonstrated a significant association between these biomarkers, highlighting their connection in the context of allergic asthma and HFrDs [41].

In our study, aortic wall thickness was significantly elevated in the HFrD groups compared to the standard diet groups, suggesting that HFrD administration, consisting of 30% fructose in drinking water, may contribute to the structural remodeling of the aortic wall. These changes could potentially play a role in the development of atherosclerotic plaque formation. Notably, these results provide evidence that HFrDs significantly influence both the morphology of the arteries and the relaxation induced by rosuvastatin, findings that are consistent with previously reported results [42,65,72,73].

As shown in our research, recent studies suggest a notable connection between elevated levels of IgE and cardiovascular health, though the mechanisms remain complex and not fully understood [74]. Increased IgE levels, often associated with allergic conditions, have been linked to a higher risk of cardiovascular diseases, including coronary heart disease, myocardial infarction and stroke [17,75,76,77]. A study analyzing data from the National Health and Nutrition Examination Survey (NHANES) found that higher total serum IgE levels were associated with increased cardiovascular mortality. In particular, individuals with elevated IgE had a 3.19-fold increased risk of cardiovascular death compared to those with lower IgE levels [78]. Additionally, a study examining both antigen-specific and total IgE levels in adults revealed that elevated serum IgE was positively linked to coronary heart disease and angina, independent of traditional cardiovascular risk factors [79]. This relationship may be driven by the inflammatory properties of IgE, which can trigger mast cell degranulation and the release of vasoactive substances that promote endothelial dysfunction and thrombosis, both of which are critical in the pathogenesis of cardiovascular disease [80].

IgE-triggered mast cells and eosinophils are proposed as pivotal contributors [17]. These cells produce chemokines and cytokines, including IL-4, IL-6, IL-9, IL-17A, IL-33, interferon-γ and TNF-α, which increase the vascular permeability and promote LDL-C and inflammatory cells infiltration into the arterial wall [81]. Research indicates that mast cells accumulate in the injured endothelium in excess, promoting foam cells’ development and atherosclerotic plaques’ formation [74,82,83]. They also play a critical role in plaque degradation and the initiation of coagulation through cytokines and proteolytic enzymes [74]. IL-6 is a pivotal cytokine in the T1-type atherosclerotic process, as it directly inhibits T-regulatory cells, thereby promoting the onset of the T1-type proatherogenic response. Additionally, IL-6 contributes to endothelial dysfunction, the initial step in vascular injury and atherosclerotic plaque evolution, and it also promotes inflammation [81,84,85,86].

Future research should focus on the role of immune cells, such as mast cells and eosinophils, in vascular dysfunction associated with allergic asthma. Investigating the effects of pro-inflammatory cytokines like IL-4, IL-6 and TNF-α could provide deeper insights into the mechanisms driving vascular remodeling. Additionally, exploring how these immune responses contribute to endothelial dysfunction and vascular injury may help clarify the relationship between allergic asthma and cardiovascular disease. Such studies could also uncover potential therapeutic strategies to prevent cardiovascular complications in allergic asthma.

Given the numerous interconnections between allergic asthma and vascular dysfunction, including inflammation, obesity, lifestyle, immunity and genetics, effectively managing both conditions may help lower systemic inflammation and, in turn, reduce the risk of cardiovascular events. For individuals with both conditions, a holistic treatment approach that targets inflammation, such as incorporating statins, can be essential for reducing long-term cardiovascular risk and may also help to slow the progression of asthma.

## 5. Conclusions

In conclusion, our study draws attention to the detrimental impact of HFrDs on metabolic and inflammatory conditions such as hyperglycemia, dyslipidemia and obesity, while shedding new light on its role in exacerbating allergic asthma. Rosuvastatin emerges as a promising therapeutic agent, not only improving lipid profiles but also reducing inflammation, improving vascular reactivity and ameliorating structural changes in both aortic and bronchial walls. These findings suggest that rosuvastatin may provide benefits in managing the overlapping pathologies such as atherosclerosis and allergic asthma, offering a potential new strategy for addressing these health issues.

## Data Availability

The original contributions presented in the study are included in the article, further inquiries can be directed to the corresponding author.

## Supplementary Materials

The following supporting information can be downloaded at: https://www.mdpi.com/article/10.3390/nu16234104/s1, Figure S1: Illustration of the organ bath myography system designed for aortic ring studies. Created with BioRender.com.

## Appendix A

**Table A1:** Description of standard rat chow according to manufacturer specifications by INCDMI “Cantacuzino” (Bucharest, Romania).

Category	Nutrient	UM	Quantity
General	Energy	Kj/g	16.00
	Fat	g/kg	50.00
	Crude protein	g/kg	180.00
	Arginine	g/kg	4.30
	Asparagine	g/kg	4.00
	Glutamic acid	g/kg	40.00
	Histidine	g/kg	2.80
	Isoleucine	g/kg	6.20
	Leucine	g/kg	10.70
	Lysine	g/kg	9.20
	Methionine + Cystine	g/kg	9.80
	Phenylalanine + Tyrosine	g/kg	10.20
	Threonine	g/kg	6.20
	Tryptophan	g/kg	2.00
	Valine	g/kg	7.40
Minerals	Calcium	g/kg	5.00
	Magnesium	g/kg	0.50
	Chlorine	g/kg	0.50
	Phosphorus	g/kg	3.00
	Potassium	g/kg	3.60
	Sodium	g/kg	0.50
	Copper	g/kg	5.00
	Iodine	g/kg	0.15
	Iron	g/kg	35.00
	Selenium	g/kg	0.15
Vitamins	Thiamine (B1)	mg/kg	4.00
	Riboflavin (B2)	mg/kg	3.00
	Pyridoxine (B6)	mg/kg	6.00
	Cyanocobalamin (B12)	mg/kg	50.0
	Menadione (K)	mg/kg	1.00
	Retinol (A)	mg/kg	1.20
	Cholecalciferol (D3)	mg/kg	25.00
	Tocopherol (E)	mg/kg	27.00
	Choline	mg/kg	750.00
