# Supplementary material for: Rosuvastatin Attenuates Vascular Dysfunction Induced by High-Fructose Diets and Allergic Asthma in Rats
Source: Nutrients. 2024 Nov 28;16(23):4104. doi: 10.3390/nu16234104 (PMC11643937; doi:10.3390/nu16234104)
Supplement: Supplementary file 1 [file nutrients-16-04104-s001.zip › nutrients-3313355-supplementary.pdf]

nutrients MDPI

Supplementary Materials

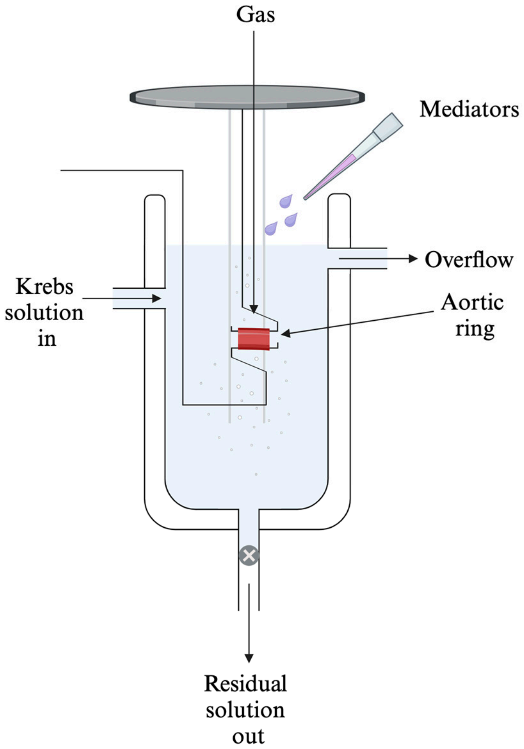

Figure S1. Illustration of the organ bath myography system designed for aortic ring studies. Created with BioRender.com.
